# Time is of the essence: past selves are not prioritized even when selective discrimination costs are controlled for

**DOI:** 10.1007/s00426-022-01702-x

**Published:** 2022-07-08

**Authors:** Julia Englert, Karola von Lampe, Nexhmedin Morina

**Affiliations:** Department of Clinical Psychology and Psychotherapy, Faculty of Psychology, University of Münster, Fliednerstraße 21, 48149 Münster, Germany

## Abstract

The perceptual Self-Prioritization effect (SPE) refers to an advantage in attending to stimuli associated with the self relative to those associated with another individual. In the perceptual matching task, arbitrary pairings between oneself and other persons, and a geometric shape need to be learned. Apart from the SPE, this task also produces high matching performance for a close other. While cognitive representations of past selves are sometimes viewed as resembling that of an intimate other, and while there is some evidence that other types of psychological closeness modulate the SPE, it remains unclear whether such prioritization effects extend to past selves. Previous experiments on this topic required participants to distinguish between different points in time within the same task, raising the possibility that potential past self-prioritization was masked by task difficulty. In our experiment, we addressed this potential confound by presenting *N* = 118 participants with a simpler version of the matching task. We re-investigated self-prioritization in perceptual matching under conditions of mental time travel to the past. In line with previous evidence, we found clear prioritization of present selves, which was evident in response times, accuracies and the efficiency of practice. Performance was consistently poorest for the past self, indicating not only a lack of privileged processing, but rather a relative de-prioritization. Performance was not improved by either increased proximity of the time period in question, nor by experimenter-induced re-imagining of the self. Our results do not support a perceptual prioritization of past selves.

## Introduction

Self-involvement modifies human information processing in a number of remarkable ways (Sui & Humphreys, 2015a). Self-prioritization extends even to arbitrary stimuli. For instance, the self-prioritization effect (SPE) in perceptual matching involves a facilitation of responses to a mapping between two stimuli, when one of those stimuli refers to the self (Sui et al., 2012). This results in an advantage in response times and accuracy for the classification of pairings of geometric shapes with a written label (perceptual matching task), given that this label refers to the self. Importantly, smaller advantages are found when the label refers to a close other, such as participant’s mother. Self-prioritization is often explained in terms of heightened perceptual salience (Humphreys & Sui, 2016; Sui & Humphreys, 2015a; Sui et al., 2014, 2015), resulting in easier attentional orienting and more efficient processing of those stimuli. The SPE is only one of a wide array of phenomena in which self-involvement affects information processing, encoding and performance. Other such effects have been demonstrated in the domains of perception and attention, memory, judgment and evaluation (Alexopoulos et al., 2012; Beggan, 1992; Brédart et al., 2006; Conway & Dewhurst, 1995; Cunningham et al., 2008; Englert & Wentura, 2016; Falbén et al., 2020; Golubickis et al., 2021; Krigolson et al., 2013; Sedikides et al., 2016; Symons & Johnson, 1997; Turk et al., 2011; van den Bos et al., 2010; Wang et al., 2019). Effects of self-relevance appear to be affected by different mechanisms. For example, self-biases in trait-descriptiveness tasks and explicit memory have been linked to later stage, more reflective, involving recollection and semantic processing (Englert & Wentura, 2016; Kotlewska & Nowicka, 2016; Wisco, 2009), while the SPE, as well as effects of the self on face processing, sustained attention, and response inhibition have been linked to earlier stage, more reflexive processes (Alexopoulos et al., 2012; Golubickis et al., 2021; Kotlewska & Nowicka, 2015; Sui & Humphreys, 2015a). This heterogeneity across existing research suggests that the term “self” itself can be divided into multiple component facets or related constructs. Multiple taxonomies for conceptualizing research on the self have been proposed (Hommel, 2018; James, 1890; Klein, 2012; Neisser, 1988; Prebble et al., 2013; for an overview of relevant terminology, see Morin, 2017).

Prebble et al. (2013) propose time and its expansion as a crucial dimension in their taxonomy of the self. Their framework builds upon the distinction between “I-self” (i.e. the self as an acting and perceiving subject) and “me-self” (i.e. the content of the self, or the self as knowledge structure that can be represented and accessed like other concepts; e.g. Kihlstrom et al., 2003) first formulated by James (1890). Prebble et al. suggest that each of the two facets can be further divided into the self at the present moment, and a temporally extended self. A subjective sense of self in the present is assumed to underlie both present self-awareness and the ability to project a subjective sense of self into either the past or future, for example during recollective experience. This immediate self-experience also supplies the “me-self” with information, allowing it to eventually “crystallize” into a temporally extended self-concept and a continuous autobiographical narrative. While the question of whether or not we “truly” remain the same person over time is complicated (Gallois, 2016; Olson, 2021), a sense of such continuity appears to serve important psychological functions (Bluck & Liao, 2013; Chandler et al., 2003). However, despite our experience of continuity in both our subjective sense of self and our narrative self-concept (Bluck & Alea, 2008; Conway et al., 2004), we also undeniably experience change (Greve et al., 2005; Hanko et al., 2009). In a sense, we can grow apart from our previous self over time, to the point where past selves can come to be represented in a manner quite similar to other persons (Pronin & Ross, 2006). This leaves open the question of how far such distancing goes. Can past selves become as distant as a stranger or passing acquaintance, or are they more akin to someone we are close to? There is some evidence that retrospective self-referential processing involves similar neural processes as other-referential processing of an intimate, rather than a merely familiar other (D’Argembeau et al., 2008; Kotlewska & Nowicka, 2015, 2016). Furthermore, it appears that certain self-processing biases, for example in explicit memory, are modulated by social closeness, with similar, albeit smaller memory advantages for close others (Symons & Johnson, 1997). A graded performance pattern is also observed in perceptual matching, where performance for an intimate other, such as participants’ mother, is generally higher than for an acquaintance, a stranger, or an object (Sui et al., 2012). There is additional evidence of individual differences in prioritization of others, which correlate with perceived social distance (Moseley et al., 2021). Since some amount of perceptual prioritization appears to be afforded by social closeness and past selves may be represented similarly to known others (Kotlewska & Nowicka, 2016; Pronin & Ross, 2006), this prompts the question if past or hypothetical future selves would likewise benefit.

Construal-Level-Theory (CLT; Trope & Liberman, 2010) provides a framework for conceptualising psychological distance, and draws parallels between various dimensions, including time and social closeness, using the self as a central reference point. Its basic assumption is that while only the present self can be directly experienced, we successfully engage in the mental construction of other objects, such as people or time periods. The key dimension along which those objects vary is to what extent we experience them as distant from our immediate self. In this view, different forms of distance—such as social, temporal or spatial—all affect this experience in a similar manner, and should have similar cognitive effects. Indeed, several studies support the notion of a common core shared by different types of psychological distance (Bar-Anan et al., 2007; Fiedler et al., 2012; Parkinson et al., 2014). If psychological distance is dimensional, then different mental objects can be ordered along a scale, preserving their relative distance to each other as well as to the self. CLT’s assumption of commensurability between different types of psychological distance lends itself to the prediction that temporally extended selves should be represented along the same scale of psychological distance as other people or objects. Increasing temporal distance should move the past self away from the intimate, toward the merely familiar (Pronin & Ross, 2006), and thus should affect self-processing biases in a similar way as social distance manipulations. The remoteness of a time period might be expected to modulate the SPE in much the same way as the remoteness of a relationship (e.g. mother vs. third cousin) does. Of course, passage of time is far from the only factor influencing identification with, or closeness of, a past self. For example, encouraging abstract, conceptual representations of a target has been shown to increase perceived distance (Stephan et al., 2011). Conversely, autonoetic consciousness (Tulving, 2002), as occurs when recollecting sensory detail from a previous experience, can reduce psychological distance. Indeed, a loss of episodic detail and increased semantization over time, as well as a shift from first person to third person perspective, are typical of memories as they become more distant (Prebble et al., 2013; Sutin & Robins, 2008). However, in some cases distant events are experienced as though they are happening “here and now”, as is the case for traumatic memories (Brewin, 2014; Ehlers & Clark, 2000), or self-defining autobiographical episodes (Singer et al., 2013), which are associated with increased sensory detail, re-experiencing and an egocentric perspective. It is therefore plausible that encouraging recollective experience and first-person perspective-taking could reduce psychological distance for a past self.

Some forms of psychological distance correlate with both the SPE and effects of self-reference in explicit memory. In the perceptual matching paradigm, intermediate performance levels are obtained for close social others, such as one’s mother, which differs both from the “self” and “stranger/acquaintance/object” conditions (Sui et al., 2012). Similarly, in the self-reference paradigm, choice of control condition is vital to the size of self-memory advantages, with smaller effect sizes when memory performance for the self-reference condition is contrasted with that for close others as compared to another person that is merely acquainted (Symons & Johnson, 1997). During trait-descriptiveness ratings, differences in strength of the late positive component (LPC) have been observed, with the strongest enhancement for the self- and smaller advantages for a close other, as compared to a non-intimate other (Kotlewska & Nowicka, 2016). Furthermore, in-group biases have been observed both in explicit reference and incidental tasks (Jeon et al., 2021; Johnson et al., 2002; Turk et al., 2008). Self-concept centrality of chosen labels, which might be considered to constitute a type of psychological distance, has been shown to mediate perceptual prioritization (Golubickis et al., 2020). This raises the possibility that the similarly graded patterns of outcomes in perceptual matching and explicit memory tasks reflect an overlap in the processes producing performance advantages for both the self and intimate others. For instance, they might reflect the large extent of knowledge about, and high personal relevance of, information pertaining to ourselves and as intimate others. In this view, performance for a past self should remain higher than performance for an acquaintance, even as the past becomes more distant, since we should retain a considerable degree of familiarity. This is in line with Kotlewska and Nowicka’s (2016) findings during self- and other-referential processing, where a past self most closely resembled an intimate other, since the LPC is associated with recollection and amount of information retrieved from memory (Vilberg et al., 2006).

Taken together, a sufficiently “close” past self may be prioritized in the perceptual matching task. Golubickis et al. (2017) addressed precisely this question by combining perceptual matching with mental time travel. In a series of three experiments, they varied temporal construal of the self, including both temporally close (yesterday, in a day) and remote (“self in 40 years”) conditions. The mental time travel conditions replaced the usual intimate other (e.g., “mother”) and were learned in addition to a present self and a stranger condition. Temporal distance to the present self was varied within participants, resulting in participants discriminating between three different points in time, and another person. The results revealed no evidence of any perceptual prioritization of either past or future selves, as compared to another person, in either of their three experiments. An SPE was only obtained for the present self. Using drift–diffusion modelling (Ratcliff & McKoon, 2008), Golubickis et al. (2017) found higher drift rates for the present self, indicating faster information uptake. Indeed, information processing appeared to be slowest under mental time travel conditions, with no differences between the various points in time. At first glance, this appears to provide a firm answer to the question of whether past selves can be perceptually prioritized either akin to the present self, or to an intimate other. Recall that the absence of any effect of temporal distance seems at odds with the view that perceptual prioritization is mediated by psychological distance,^1^ while the lack of prioritization of past selves suggests that, for the purposes of perceptual matching, past selves are not represented like close others. This would seem in tension with CLT’s (Trope & Liberman, 2010) view that temporal and social distance function analogously in cognition.

We believe, however, that Golubickis et al.’s (2017) experimental design poses one important limitation. In a typical perceptual matching set-up, only three shape-label combinations need to be learned distinguished from each other. Yet, in Golubickis et al.’s design, participants were required to learn four such pairings, increasing cognitive demand. There is reason to believe that this four-level design worked to selectively impair performance in the mental time travel conditions. Rather than simply representing a past or future self, two such “selves” needed to be distinguished. Each participant had to discriminate between the present self, a stranger and either two different future selves or two different past selves. Arguably, in each given task, those two mental time travel conditions shared the most similarity with each other, making them more difficult to discriminate. Therefore, varying levels of temporal distance within participants might have created a challenge unique to the mental time travel conditions, imposing a selective cost on only those conditions. Such a selective discrimination cost could in turn have cancelled out, and thereby masked, a potential prioritization of past selves. This is especially plausible since the SPE obtained by Golubickis et al. was driven by differences in drift rates, which have been shown to be lower in a discrimination task when stimuli are more difficult to distinguish (Voss et al., 2004).

To address this alternate possibility, we combined the perceptual matching paradigm with mental time travel, reverting to a simpler three-level design. Each participant was required to discriminate only between the present self, a stranger and one past self condition. Temporal distance was varied as a between group factor. Since time alone may not be the best indicator of psychological distance, we also varied mental time travel instructions: Half of participants were given an instruction to induce autonoetic remembering of the time period in question, to encourage taking the perspective of the past self, and to render the past self-representation more accessible. As a manipulation check for this induction, we included a self-report measure for participants’ subjective experience of the past self, based on the Memory Experiences Questionnaire (MEQ; Sutin & Robins, 2007). If cognitive representations of past selves qualitatively resemble those of close others, we can predict that the past self-label would be associated with intermediate performance levels as well. That is, performance for past selves should be poorer relative to present selves and higher relative to strangers. If psychological distance underlies relative prioritization and is reduced by following instruction to reminisce, receiving such instructions should lead to better matching performance for past selves. Furthermore, if matching performance is a linear function of psychological distance, and if temporal remoteness serves as a reliable proxy for this distance, a temporally close self should be more likely to be prioritized than a temporally distant one. On the other hand, the effects of mental time travel on perceptual matching might be categorical, with no differences in effect sizes based on relative temporal distance. No such covariation between temporal distance and matching performance occurred in the previous experiments manipulating this factor (Golubickis et al., 2017). Even if past self-prioritization was obscured by selective discrimination costs, it is not clear why such costs should cancel out differences between nearer and more remote past selves. Therefore, this potential confound cannot readily account for a lack of modulation of matching performance by temporal distance. While we vary temporal distance between groups, the primary focus of this experiment is on whether any prioritization of the past self occurs at all.

## Method

### Sample and participants

To avoid the problem of insufficient test power (Szucs & Ioannidis, 2017), we based our power calculation on the smallest effect size of interest obtained in Englert’s (2018) Experiment 7, that is, the reaction time difference between the “self” and “mother” conditions in matching trials (*d*_z_ = 0.33). Using the GPower tool (Faul et al., 2007), we estimate that *N* = 59 participants are required to detect an effect of *d*_z_ = 0.33 with an accepted type one error *α* = 0.05 (two-tailed) and a power 1-*β* = 0.80. Based on our theoretical reasoning, the past self should resemble an intimate other, and therefore, the past self condition conceptually corresponds most closely to the “mother” condition in previous perceptual matching studies.^2^ However, since we are interested in whether or not any prioritization of the past self can be observed, the critical comparison is between the past self and the stranger condition. For this comparison, effect sizes in Englert (2018) were *d*_z_ = 0.81 and *d*_z_ = 0.91, for accuracies and reaction times, respectively. For either effect, test power would be 1-*β* > 0.99 given *N* = 59. Since we hypothesized that perceptual prioritization might be moderated by the remoteness (1 day vs 5 years) of the past self, we aimed for double the number of observations,^3^ resulting in the aforementioned power estimates applying to either group independently.

A secondary focus of our experiment were potential effects of remoteness. To evaluate our design’s suitability for this purpose, we calculated minimum effect sizes that would have been detectable with a power of 1-*β* = 0.8 given our sample size of *N* = 118. An effect of temporal distance could either be expressed as a 2 × 3 interaction between the factors remoteness and referent, or, since prioritization should be relative to the stranger condition, as a 2 × 2 interaction between remoteness and the past self and stranger referents only. Alternatively, one could predict a mean group difference between the close and remote past self conditions. We used the Superpower R-package (Lakens & Caldwell, 2021) for a post hoc calculation of the minimum effect size required for such 2 × 3 or 2 × 2 interactions, and GPower to do the same for a between-group mean difference. Assumed correlations between dependent measures corresponded to the empirical correlation in the present experiment (*r* = 0.43^4^). Given *α* = 0.05, we would have been able to detect an interaction effect of Cohen’s *f* = 0.14 for the 2 × 3 interaction, and of *f* = 0.18 for a 2 × 2 interaction. By convention, this corresponds to a small to medium effect size. We would have been able to detect a between-group difference of Cohen’s *d* = 0.52 with a power of 1-*β* = 0.8. By convention, this corresponds to a medium effect size.

The link to our study was opened 731 times and 132 people completed the study. No further information on non-completing participants is provided here, since we took the prudent stance of interpreting early termination as withdrawal of consent from the experiment (British Psychological Society, 2017). We excluded 14 participants based on their diligence as indicated by performance and self-report: Of those, one participant indicated not having properly followed the instructions, seven participants failed to produce at least 50% valid responses, and six participants had 50% or more errors among their valid responses. Thus, *N* = 118 participants (72f, 46 m, median age = 24 years, ranging from 18 to 59 years) were included in the analysis. Recruitment took place online, via virtual bulletin boards and mailing lists at the university, as well as via advertisements in social media. Psychology students at the University of Münster received 0.75 h of course credit for their participation. At the end of the experiment, participants were offered the opportunity to take part in a lottery for 13 monetary prizes, totalling 200 Euros.

### Design

The experiment followed a 2 × 2 × 3 × 2 design, with the factors remoteness of past self (yesterday/5 years ago) and reminiscence instructions (present/absent) varied between participants, and the factors referent label (present self/past self/stranger) and matching condition (matching/non-matching) varied within participants. Main outcome measures were matching accuracy and reaction times. We additionally assessed subjective mnemonic experience using a five-point rating scale.

### Materials and apparatus

#### Hardware and software

The study was conducted online,^5^ requiring participants to have a desktop or laptop computer, with a screen, keyboard and mouse. For data security reasons, demographic information was collected at the beginning, using the Unipark platform (Questback GmbH et al., 2017) and stored separately on the provider’s secure server. All other data were collected and stored using the Labvanced experiment creation software (Finger et al., 2017). To connect the data sets while guaranteeing anonymity, participants generated an eight-digit personalized code that they provided in both sections of the study.

#### Matching stimuli

For the matching task, we used three .png image files, which were 118 × 118 pixels in size,^6^ with the size of the respective geometric shapes (Circle, Square, and Triangle, see Appendix 1) taking up a maximum of the area. The shapes were opaque blue (Hex: #4472c4). For the circle and triangle, a plain white background remained. Labels consisted of the words “I now”, “I then” and “a stranger” in black capital letters (Font: Lato, Font Size: 48).

#### Self-report questions

Questionnaire items were based on the Memory Experience Questionnaire’s (MEQ; Sutin & Robins, 2007). The subscales we adapted as basis for our questions were Vividness, Accessibility, Sensory Detail, Emotional Intensity, Visual Perspective, Distancing, and Valence. We made a priori decisions about the suitability of the respective questions to our task, and rephrased some of the items so they referred to the past self in our matching task, rather than to a specific autobiographical episode, as is the case in the original questionnaire. The questions we used as well as their English translations are listed in Appendix 1.

### Procedure

Participants started each session by following a link to the Unipark platform where they were welcomed, provided with information about the experimental procedure and the use of their data, and then offered the opportunity to either give informed consent or abort. Afterwards, they were asked to generate their personalized eight-digit code, and to provide information about their age, gender, handedness, German language proficiency, and professional status. Consenting participants between 18 and 59 years of age who indicated to be at least “fluent” in German were then redirected to Labvanced for the second part of the experiment. This section consisted of a learning phase, a practice matching block, four test matching blocks, and the self-report questionnaire. After entering their personalized code a second time, participants were briefed on the remaining procedure.

**Fig. 1:**
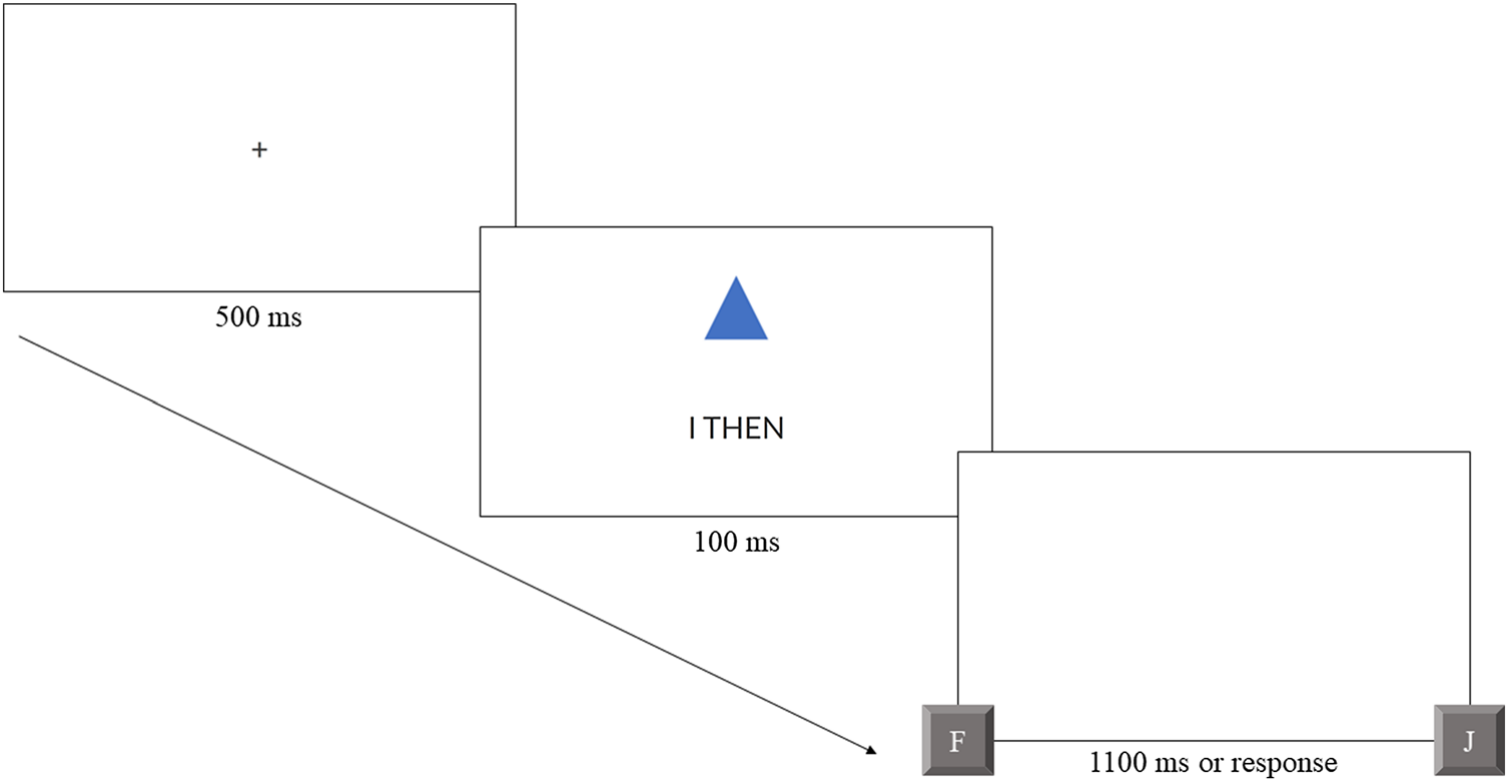
Exemplification of a perceptual matching trial in the test phase, depicting the remote past self condition. Participants judged whether the shape-label pairing matched the pairing they had previously learned (“J”) or not (“F”)

At the start of the learning phase, half of participants were instructed to recollect their past self (induction vs. no induction), imagining themselves either as they were yesterday, or 5 years ago from a first-person perspective. The duration of this phase was self-paced. Participants were asked to continue via the Space key once they had a clear idea of their past self. Then, all participants received matching task instructions: they were asked to memorize three pairings of labels pertaining to either their present self, their past self, or a stranger, and either a square, round or triangular geometric shape. For half of participants, the “past self” label referred to themselves a day ago (“I yesterday”) and for half of participants, it referred to themselves 5 years ago (“I then”). This time period always matched the reminiscence instructions for those participants who had received them. The pairings were presented in text form on screen for a duration of 60 s, during which each participant had time to learn an unambiguous assignment between each of the labels and shapes. For example, a particular assignment could have read “I now am the CIRCLE. I then am the SQUARE. A stranger is the TRIANGLE”. Assignments were counterbalanced across participants according to a Latin square, and were orthogonal to the remoteness and instruction factors. The learning phase was followed by the matching task. An illustration of the matching trial procedure is shown in Fig. 1. Participants were instructed to respond as fast and as accurately as possible, to label-shape pairings shown on screen, by pressing either “J” if a given pairing corresponded to the assignment they had just learned (matching trial), and by pressing “F”, if it did not (non-matching trial). Participants could then start a practice block to familiarize themselves with the matching task and rehearse the assignments. All stimuli were presented on a white background. At the beginning of each practice trial, a black fixation cross appeared at the centre of the screen for 500 ms.^7^ This was followed immediately by the presentation of the shape-label pair, which lasted for 100 ms. Shapes were presented slightly above, labels slightly below the center of the screen. The shape-label combination was replaced by a blank screen for 1100 ms during which participants had the opportunity to give a matching or non-matching keypress response. For the practice trials alone, a feedback screen was shown to participants for 4000 ms each time after those 1100 ms had expired. Feedback was provided in black letters (Font: Lato, Font Size: 28) at a center-top position of the screen. If participants responded within the response window, they learned whether their response was correct or incorrect. If they did not respond in time, they were told that they had been too slow and asked to respond within one second. Matching assignments were simultaneously repeated below the feedback. Each participant worked through 18 practice trials. During the practice block, each possible shape-label pairing was presented twice, resulting in 6 matching, and 12 non-matching trials in a random order.

**Fig. 2:**
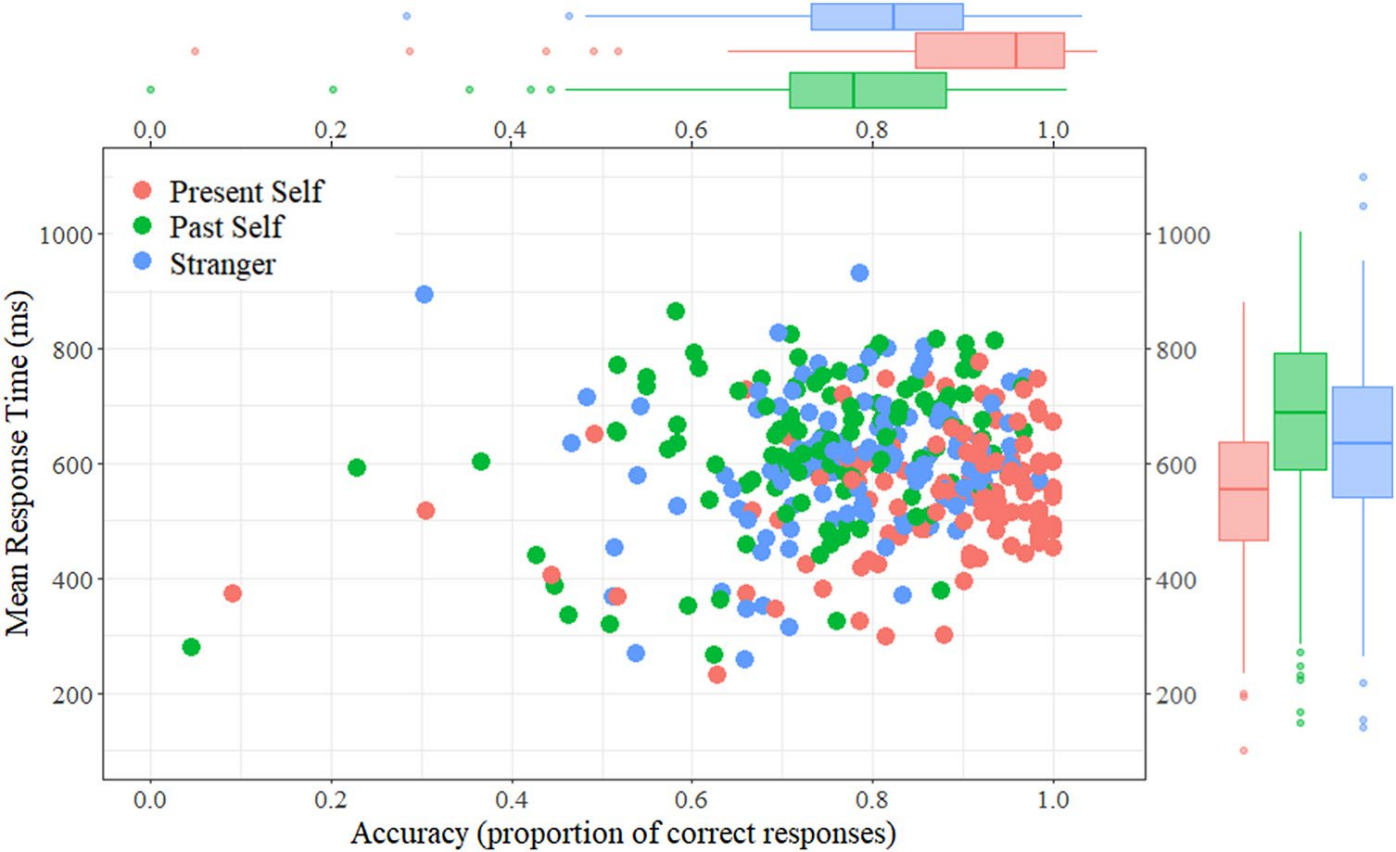
Matching effects for the present self, past self and stranger condition in matching trials. We depict the distribution of condition means for each participant’s performance, for matched stimulus pairs. The x-axis represents the proportion of correct responses and the y-axis represents reaction times. For the boxplots, horizontal lines show the median, while the length of the boxes represents the interquartile range, and the tails exclude outliers. Note. This mode of visualization retains interindividual variance that is controlled for in our statistical tests for dependent measures

After the practice block, participants were informed about the approximate duration of the following phase and received a refresher on the matching task instructions, before starting the test phase by pressing space. The test phase consisted of four blocks of the perceptual matching task, each lasting approximately 3 min. Test trials followed the same procedure as the practice trials, with one exception: No more feedback slides were shown. The task, as well as the label-shape and response assignments remained the same. An illustration of the trial procedure is shown in Fig. 2.

Participants worked through four blocks of 96 test trials each, adding up to a total of 384 trials. Between each block, they had the opportunity to take a self-paced break. During each break, a progress bar, as well as reminders of label-shape and response key assignments were presented on screen. For each test block, matching and non-matching trials occurred equally often, resulting in 16 repetitions of each matching, and 8 repetitions of each non-matching combination in randomized order.

After the matching task was over, participants gave self-reports on their mnemonic experience during the experiment. Participants were explicitly instructed to answer based on their subjective experience of the past self and its corresponding time period as referred to by the experimental instructions. Participants rated their agreement with each of the twenty questionnaire items on a five-point scale, ranging from 1 (“disagree completely”) to 5 (“agree completely”). Statements were presented in the same order for all participants. Finally, participants were thanked for their participation, asked a control question about their understanding and diligence during the experiment, and provided with information for redeeming their course credit, survey circle code, and contacting the authors.

## Results

### Treatment of data and statistical analysis

Unless otherwise noted, all effects referred to as statistically significant throughout the article are associated with *p*-values of < 0.05, two-tailed. Analyses employed for the purpose of statistical inference are described alongside their respective results.

Only participants who indicated that they had taken the task seriously and who had made at least 50% valid responses during the matching task, of which at least 50% were correct, were concluded in the analysis.^8^ No valid responses were recorded for 8.47% of all trials (3858 trials total). Response times were recorded from the offset of the shape-label pair. Responses made faster than 100 ms after stimulus offset were excluded from the analyses. As an upper bound, we used the Tukey criterion for outliers, resulting in a cut-off value of 1126 ms,^9^ and the exclusion of one trial. Mean reaction times and accuracies by referent label and remoteness of past time period are shown in Table 1. For the self-report measures, subscales were recreated based on the respective subscale of the MEQ the original items belonged to. For those subscales achieving satisfactory internal consistencies (defined as Cronbach’s *α* < 0.6), mean agreement was computed. Self-report means by remoteness of past time period and instruction condition are shown in Table 2. An overview of all items, subscales and internal consistencies is provided in Appendix 1.

### Matching performance

#### Matching trials

Mean reaction times and accuracies are shown in Table 1. In a first step, we tested for perceptual prioritization of either present or past selves in general. Since our main aim was to determine whether any matching advantage would occur for the past self over a stranger, we did not include either of the group factors in this initial analysis (for a breakdown of results by remoteness and induction, see the next section “Role of Remoteness and Induction on Mnemonic Experience and Matching Performance”).

**Table 1:** Mean reaction times (in ms) and accuracies (in %) as a function of remoteness group (near past/“yesterday” and remote past/ “5 years ago”, referent label (current self, past self and stranger) and trial type (matching and non-matching)

Remoteness group	Referent	*N*	Reaction time		Accuracy	
			Matching	Non-matching	Matching	Non-matching
Near past	Present self	58 60	538 (100) 553 (107)	655 (111) 669 (125)	.85 (.17) .89 (.10)	.75 (.16) .73 (.17)
Remote past						
Near past	Past self	58 60	619 (131) 632 (118)	649 (115) 672 (118)	.73 (.16) .75 (.14)	.75 (.15) .73 (.15)
Remote past						
Near past	Stranger	58 60	588 (115) 614 (121)	633 (109) 650 (112)	.80 (.12) .76 (.12)	.85 (.12) .83 (.14)
Remote past						

SD in parentheses

To test for perceptual prioritization of either present or past selves, we conducted separate repeated-measures analysis of variance (ANOVA) on reaction times and accuracies for the matching trials only, with referent label (present self, past self, stranger) as a within subjects-factor. To test our hypotheses regarding the graded performance pattern, planned Helmert contrasts were computed between the present self and the combined past self/ stranger conditions, as well as between the past self and stranger condition. There were main effects of label type on both mean reaction times (*F*_2, 234_ = 97.25, *p* < 0.001, partial *η*^2^ = 0.454) and mean accuracies (*F*_1.84, 215.43_ = 49.41, *p* < 0.001, partial *η*^2^ = 0.297). As predicted, performance was highest in the present self, as compared to the past self and stranger conditions (*F*_1, 117_ = 157.68, *p* < 0.001, partial *η*^2^ = 0.574, for reaction times, *F*_1, 117_ = 9.94, *p* < 0.001, partial *η*^2^ = 0.445, for accuracies). Contrary to our prediction, performance was higher in the stranger condition than for the past self conditions, in both reaction times (*F*_1, 117_ = 19.89, *p* < 0.001, partial *η*^2^ = 0.145) and accuracies (*F*_1, 117_ = 8.99, *p* = 0.003, partial *η*^2^ = 0.07). The distribution of participant’s mean performance for the present self, past self, and stranger conditions during matching trials is depicted in Fig. 2.

#### Non-matching trials

Mean reaction times and accuracies are shown in Table 1.

**Fig. 3:**
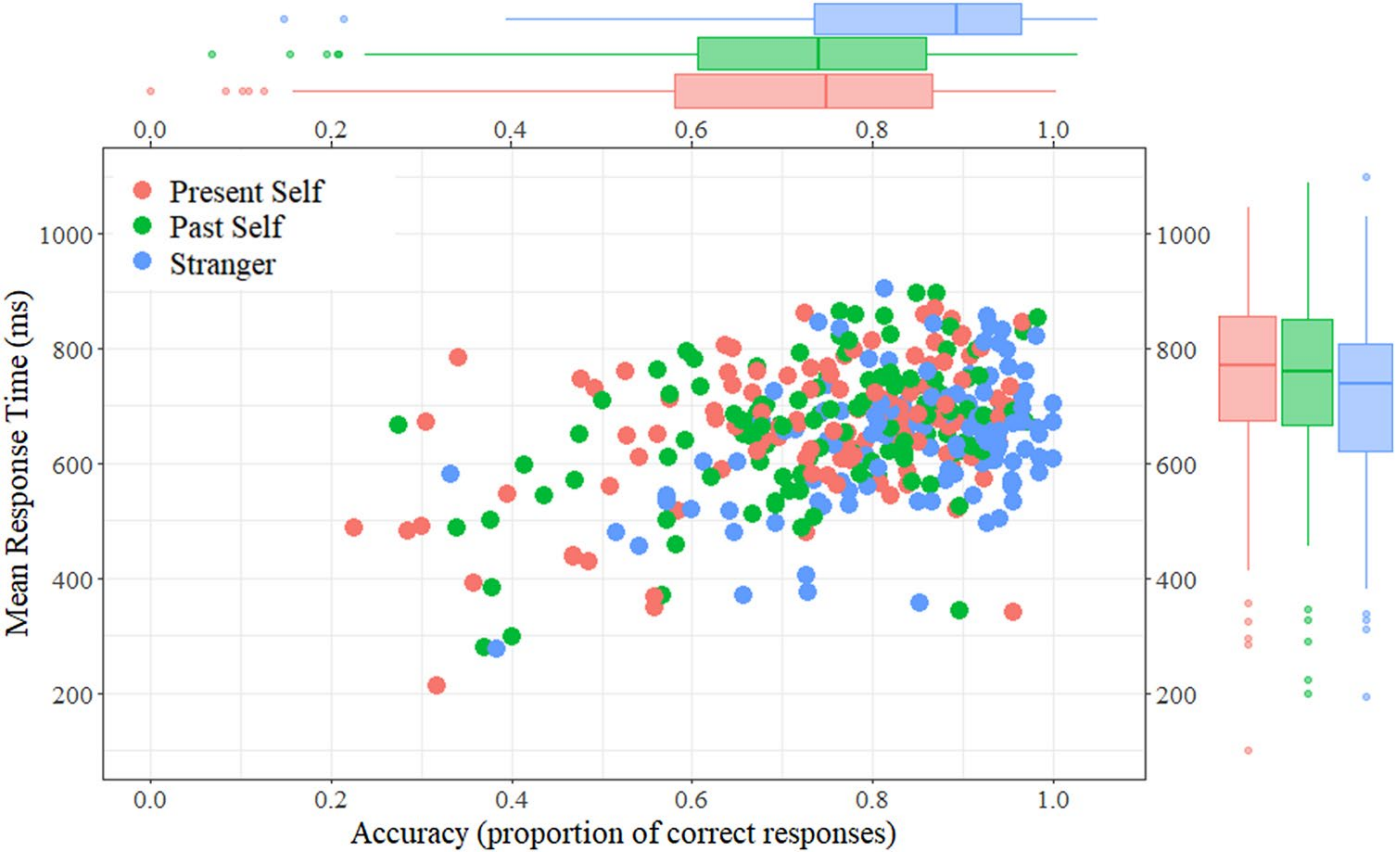
Matching effects for the present self, past self and stranger condition in non-matching trials. We depict the distribution of condition means for each participant’s performance, for non-matching stimulus pairs. The *x*-axis represents the proportion of correct responses and the *y*-axis represents reaction times. For the boxplots, horizontal lines show the median, while the length of the boxes represents the interquartile range, and the tails exclude outliers. *Note* This mode of visualization retains interindividual variance that is controlled for in our statistical tests for dependent measures

Since the SPE appears to be most robust and consistent in matching trials (Schäfer et al., 2015; Sui et al., 2012),^10^ our main predictions concern the matching trials. However, we also report the non-matching trials, based on two separate repeated-measures ANOVAs for reaction times and accuracies with referent label (present self, past self, stranger) as a within subjects-factor. The main effect of referent label was significant for both reaction times (*F*_2, 234_ = 18.55, *p *< 0.001, partial *η*^2^ = 0.137) and accuracies (F_2, 234_ = 90.82, *p *< 0.001, partial *η*^2^ = 0.437). Bonferroni-Holm-adjusted pairwise comparisons showed that performance was highest in the stranger condition, which differed significantly from the present self (MD = 21 ms, *p *< 0.001 for reaction times, MD = 0.105, *p *< 0.001 for accuracies), and the past self condition (MD = 19 ms, *p *< 0.001 for reaction times, MD = 0.099, *p *< 0.001 for accuracies), with no statistically significant difference between the past and present self-conditions (MD = 1 ms, *p *= 0.689 for reaction times, MD = 0.006, for accuracies, *p *= 0.482). The distribution of participant’s mean performance for the present self, past self, and stranger conditions during non-matching trials is depicted in Fig. 3.

### Role of remoteness and induction on mnemonic experience and matching performance

We examined the effects of the group factors remoteness (yesterday vs. 5 years ago) and reminiscence induction (instruction vs. no instruction) on matching performance and accuracies, and on subjective mnemonic experience as measured by the MEQ. Self-report means by remoteness of past time period and instruction condition are shown in Table 2.

**Table 2:** Overview of mnemonic experience self-reports by remoteness and induction condition

Subscale	*n* (items)	Induction		No induction	
		Yesterday (*n* = 29)	5 years ago (*n* = 29)	Yesterday (*n* = 29)	5 years ago (*n* = 31)
Vividness	4	3.67 (.87)	3.16 (.80)	2.59 (.87)	2.54 (.92)
Accessibility	4	3.72 (1.01)	3.31 (1.20)	3.40 (.89)	2.77 (.96)
Valence	4	3.61 (.93)	3.82 (.71)	3.54 (.75)	3.70 (.71)
Distancing	2	2.12 (.99)	3.92 (.92)	2.50 (1.02)	3.48 (.84)

Means for the subscales with a Cronbach’s *α* > .60. SDs in parentheses

#### Mnemonic experience

For subjective memory experience, we tested internal consistencies among groups of items that corresponded to the subscales of the MEQ.^11^ Following the structure of the MEQ, our items were a priori assigned to the subscales Vividness, Accessibility, Valence, Distancing, Emotional Intensity, Visual Perspective and Sensory Detail. Items with their English translations and respective assignment, as well as Cronbach’s α value for each of those subscales are shown in Appendix 1. Since scale reliability is critical to the possibility of detecting meaningful correlations (see, e.g., Danner, 2016), only subscales reaching a Cronbach’s α of 0.6 or higher were included in further analyses, leading to the inclusion of four of the seven subscales (Vividness, Accessibility, Valence, and Distancing.)

For each of those four subscales, we conducted 2 × 2 two-way ANOVAs with remoteness (yesterday vs. 5 years ago) and reminiscence (instruction vs. no instruction) as between-subject factors, and the respective mean scale value as dependent variables. The reminiscence manipulation had significant effects on self-reports for Vividness (*F*_1, 114_ = 27.77, *p *< 0.001, partial *η*^2^ = 0.196) and Accessibility (*F*_1, 114_ = 5.28, *p *= 0.023, partial *η*^2^ = 0.044), with higher vividness and accessibility of the past self for participants who had received reminiscence instructions prior to the experiment. Remoteness of the past self had significant effects on self-reports for Accessibility (*F*_1, 114_ = 7.60, *p *= 0.007, partial *η*^2^ = 0.063) and Distancing (*F*_1, 114_ = 38.36, *p *< 0.001, partial *η*^2^ = 0.252), with participants reporting the self 5 years ago as less accessible and more distant than the self yesterday. No main effects of reminiscence instructions were observed on either Valence (*F*_1, 114_ = 0.42, *p *= 0.517, partial *η*^2^ = 0.004) or Distancing (*F*_1, 114_ = 2.68, *p *= 0.104, partial *η*^2^ = 0.023). No main effects of remoteness were observed on either Vividness (*F*_1, 114_ = 2.92, *p *= 0.09, partial *η*^2^ = 0.025) or Valence (*F*_1, 114_ = 1.63, *p *= 0.204, partial *η*^2^ = 0.014). No interactions between reminiscence and remoteness were observed on any of the subscales (F_1, 114_ = 2.02, *p *= 0.158, partial *η*^2^ = 0.017 for Vividness; *F*_1, 114_ = 0.31, *p *= 0.580, partial *η*^2^ = 0.003 for Accessibility; *F*_1, 114_ = 0.03, *p *= 0.866, partial *η*^2^ < 0.001 for Valence; *F*_1, 114_ = 2.93, *p *= 0.589, partial *η*^2^ = 0.003 for Distancing).

#### Matching performance: matching trials

For matching performance, we computed two separate mixed-models 2 × 2 × 3 ANOVAs on reaction times and accuracies for the matching trials. We included referent label (present self/ past self/, stranger) as a within subjects-factor, and remoteness (yesterday vs. 5 years ago) and reminiscence (instructions vs. no instructions) as between-subjects factors (for mean reaction times and accuracies broken down by referent condition and remoteness group, see Table 1. Appendix 2 further breaks down results by reminiscence instruction group). There was a main effect of referent condition on both dependent variables (*F*_2, 228_ = 98.60, *p *< 0.001, partial *η*^2^ = 0.464, for reaction times, *F*_1.86, 212.25_ = 50.13, *p *< 0.001, partial *η*^2^ = 0.305, for accuracies).

For accuracies, there was also an interaction between referent and remoteness (*F*_1.86, 212.25_ = 4.12, *p *= 0.020, partial *η*^2^ = 0.035). When considering contrasts between referent conditions for each remoteness group individually, the comparison between the past self and stranger conditions was no longer evident in the “5 years ago” group (*F*_1, 59_ = 0.60, *p *= 0.442, partial *η*^2^ = 0.010), while all other contrast differences were preserved in both groups (*F*_1, 57_ = 23.8, *p *< 0.001, partial *η*^2^ = 0.296 for present self vs. past self/other, “yesterday” group, *F*_1, 57_ = 10.81, *p *= 0.002, partial *η*^2^ = 0.159 for present self vs. past self/other “5 years ago” group, *F*_1, 57_ = 93.00, *p *< 0.001, partial *η*^2^ = 0.612 for past self vs stranger, “yesterday” group). To understand the source of the interaction effect, we then compared simple differences between individual referent conditions by remoteness group, controlling for α-error accumulation using Bonferroni-Holm-adjustment. Descriptively, there were smaller differences between the present self and the stranger condition (Δ*M *= 0.053, SD = 0.189 versus, Δ*M *= 0.125, SD = 0.121, t = 2.48, *p *= 0.015 unadjusted, *p *= 0.045 adjusted), and—although negligibly so—between the past and present self condition (Δ*M *= 0.123, SD = 0.163 vs Δ*M *= 0.138, SD = 0.130, t = 0.64, *p *= 0.525, unadjusted = adjusted) in the “yesterday” as compared to the “5 years ago” group, while there was a larger difference between the past self and the stranger condition (Δ*M *= 0.071, SD = 0.163 versus Δ*M *= 0.013, SD = 0.129, t = 2.12, *p *= 0.036, unadjusted, *p *= 0.074, adjusted) for the “yesterday” as compared to the “5 years ago” group. When correcting for multiple comparisons, only the group effect on the difference between the present self and the stranger condition was statistically significant. None of the group differences on accuracies for each referent individually was statistically significant (Δ*M *= -0.036; t = 1.38, *p *= 0.085 for present self, Δ*M *= -0.021, t = 0.779, *p *= 0.437 for past self, Δ*M *= 0.036, t = 1.63, *p *= 0.106 for stranger, see Table 1 for means and SDs), even without α-error adjustment.

The two-way interaction between referent and remoteness did not approach significance for the reaction times (*F*_2, 228_ = 0.702, *p *= 0.497, partial *η*^2^ = 0.006). Neither the main effects of remoteness (*F*_1, 114_ = 0.74, *p *= 0.392, partial *η*^2^ = 0.006, for reaction times; *F*_1, 114_ = 0.12, *p *= 0.729 partial *η*^2^ = 0.001, for accuracies) nor of reminiscence instructions (*F*_1, 114_ < 0.001, *p *= 0.995, partial *η*^2^ < 0.001, for reaction times; *F*_1, 114_ = 0.02, *p *= 0.878, partial *η*^2^ < 0.001, for accuracies) reached significance for either dependent variable. Neither the two-way interactions between remoteness and reminiscence instructions (*F*_1, 114_ = 0.334, *p *= 0.565, partial *η*^2^ = 0.003, for reaction times; *F*_1, 114_ < 0.01, *p *= 0.991, partial *η*^2^ < 0.001, for accuracies), nor between referent and reminiscence instructions (*F*_2, 228_ = 2.72, *p *= 0.068, partial *η*^2^ = 0.023, for reaction times; *F*_1.86, 212.25_ = 0.75, *p *= 0.466, partial *η*^2^ = 0.007, for accuracies) nor the three-way interaction between referent, remoteness and reminiscence instructions (*F*_2, 228_ = 1.29, *p *= 0.278, partial *η*^2^ = 0.011, for reaction times; *F*_1.86, 212.25_ = 0.02, *p *= 0.974, partial *η*^2^ < 0.001, for accuracies) reached significance for either dependent variable.

#### Matching Performance: Non-matching Trials

We also computed two separate mixed-models 2 × 2 × 3 ANOVAs on reaction times and accuracies for the non-matching trials, including label (present self/ past self/, stranger) as a within subjects-factor, and remoteness (yesterday vs. 5 years ago) and reminiscence (instructions vs. no instructions) as between-subjects factors (for mean reaction times and accuracies broken down by referent condition and remoteness group, see Table 1. Table B1 further breaks down results by reminiscence group). For both reaction times (*F*_2, 228_ = 18.34, *p *< 0.001, partial *η*^2^ = 0.139) and accuracies (*F*_2, 228_ = 89.59, *p *< 0.001, partial *η*^2^ = 0.440), the main effect of referent reached significance. Neither the main effects of remoteness (*F*_1, 114_ = 0.69, *p *= 0.407, partial *η*^2^ = 0.006, for reaction times; *F*_1, 114_ = 0.48, *p *= 0.491 partial *η*^2^ = 0.004, for accuracies), nor of reminiscence instructions (*F*_1, 114_ = 0.18, *p *= 0.675, partial *η*^2^ = 0.002, for reaction times; *F*_1, 114_ = 0.01, *p *= 0.919, partial *η*^2^ < 0.001, for accuracies) reached significance for either dependent variable. Neither the two-way interactions between remoteness and reminiscence instructions (*F*_1, 114_ = 0.06, *p *= 0.809, partial *η*^2^ = 0.001, for reaction times; *F*_1, 114_ = 0.13, *p *= 0.724, partial *η*^2^ = 0.001, for accuracies), referent and remoteness (*F*_2, 228_ = 0.77, *p *= 0.463, partial *η*^2^ = 0.007, for reaction times; *F*_2, 228_ = 0.03, *p *= 0.973, partial *η*^2^ < 0.001, for accuracies), nor referent and reminiscence instruction (*F*_2, 228_ = 0.40, *p *= 0.669, partial *η*^2^ = 0.004, for reaction times; *F*_2, 228_ = 1.76, *p *= 0.174, partial *η*^2^ = 0.015, for accuracies), nor the three-way interaction between referent, remoteness and reminiscence instructions (*F*_2, 228_ = 0.13, *p *= 0.880, partial *η*^2^ = 0.001, for reaction times; *F*_2,228_ = 0.18, *p *= 0.833, partial *η*^2^ = 0.002, for accuracies) reached significance.

## Discussion

We investigated self-prioritization in a perceptual matching task under mental time travel conditions in an online experiment. Our results suggest that self-prioritization is limited to the present. We found a clear SPE in both reaction times and accuracy, with superior performance for the present self condition in matching trials, in line with existing research. Specifically, the performance advantage for the present self over a stranger mirrors the SPE typically found in the literature (Englert, 2018; Schäfer et al., 2015, 2016; Sui et al., 2012, 2014). Therefore, our present self appears to be functionally similar to the self-labels in other studies on the SPE. Our findings further indicates that the matching paradigm is suitable for online experimentation.

Crucially, perceptual matching performance was lower for the past self than for both the present self and a stranger. This was largely true regardless of temporal distance^12^ from the past self or instructions to reminisce, even though our self-report measures were sensitive to those manipulations. While there was an interaction between remoteness of the past self and referent condition on matching performance, this appeared to only affect performance in the present self and stranger conditions directly. In addition, we found a seemingly reversed pattern of performance in the non-matching condition, showing better performance when rejecting the stranger label compared to either the present or past self-label.

Our theoretical rationale predicted some amount of past self-prioritization, based on psychological distance. However, if anything, past selves were de-prioritized. Therefore, the lower performance for the past self compared to the stranger condition was contrary to our hypothesis. We further included two group manipulations we hypothesized might affect psychological distance, neither of which had an effect on matching performance for the past self. First, we gave instructions to reminisce on the past self to half of our sample, expecting this to reduce psychological distance to the past self. Recollective experience and re-experiencing, which we encouraged via those instructions, are considered central to the subjective experience of the temporally extended self, and also weaken with increasing temporal distance (Prebble et al., 2013). Indeed, the experience of the past self was described as more vivid and more accessible in this group. Vividness is associated with greater perceptual detail in memory, that is, a more concrete construal, according to CLT, while accessibility refers to the ease with which a memory can be retrieved and is linked to salience and personal relevance (Rathbone & Moulin, 2014). However, at no point did we observe effects of this manipulation on matching performance. Second, we manipulated temporal distance from the past self between groups, referring to either a close (“yesterday”) or remote (“5 years ago”) past. Participants reported higher accessibility and lower distancing for a near than for a remote self, suggesting that temporal distance at least partially coincides with psychological distance. The distancing dimension on our questionnaire directly pertains to the experience of a previous self as another person (Pronin & Ross, 2006). However, past self-matching performance was the same for both groups. The absence of an effect of temporal distance on past self-matching in both our and the previous experiments (Golubickis et al., 2017) appears to suggest a disconnection between different types of psychological distance, with the social dimension apparently modulating perceptual prioritization, while the same cannot be said for the temporal dimension. This is problematic for an interpretation of the SPE in terms of psychological distance. A central assumption of CLT (Trope & Liberman, 2010) is that different types of psychological distance function analogously to each other which seems at odds with the differing effects of temporal and social distance manipulations. Specifically, if the past self could be considered in much the same way as a known other (Kotlewska & Nowicka, 2016; Pronin & Ross, 2006) for the purposes of perceptual prioritization, we would have observed an advantage for the past self over a stranger, who, by definition, and much unlike our own history, is unfamiliar to us.

However, as Trope and Liberman (2010) point out, various types of psychological distance need not be equivalent simply because they share common properties. Rather, some dimensions might take precedence over others, for example through greater availability of sensory experience. Time appears to be subordinate to space in just this manner (Boroditsky & Ramscar, 2002). If social distance was more immediate to our experience than temporal distance and therefore required less of a top-down construal process, it stands to reason that it would have a stronger impact on a fast-acting, comparatively automatic phenomenon like perceptual prioritization. For instance, while temporal distance was not found to prime spatial distance (Boroditsky & Ramscar, 2002), it has been found to do so for social distance (Stephan et al., 2011). On the other hand, while, for example, Bar-Anan et al. (2007) did obtain somewhat smaller congruency effects between spatial and temporal distance, as compared to spatial and social distance in a Stroop task, both dimensions affected attentional processes in a similar manner. Other evidence also points toward behavioral and neural commonalities between the two dimensions (Fiedler et al., 2012; Parkinson et al., 2014). Another potential caveat is that, even if social and temporal distance are commensurate, this does not tell us the location of any particular mental object with regard to its psychological distance to the self. In other words, it is unknown how exactly temporal and social distance scale in the human mind: It cannot be ruled out that, in some respects, a former self is simply experienced as more distant than a stranger.

A more trivial explanation might be that our operationalization was flawed. It is of course simplistic to view psychological distance as a linear function of the mere passage of time, and therefore, this factor might be overshadowed by other factors such as personal relevance or frequency and recency of access. Just as episodic detail typically decreases with time (Prebble et al., 2013), its retrieval or reconstruction may be encouraged through the right cues or instructions, which may then reduce psychological distance of a remote memory. However, instructing participants to reminisce on, and take the perspective of the past self did not lead to perceptual prioritization. Further avenues for addressing this concern may be a more fine-grained parametrization of the dimension in question, as well as probing psychological distance more directly and immediately (Sui & Humphreys, 2015b). When discussing this possibility, it needs to be noted that both the remoteness of the time period in question, and instructions to reminisce did affect self-reports in the expected way. This suggests that we indeed successfully manipulated psychological distance. Of course, the self-reports of participants’ mnemonic experience should be interpreted with caution. Our questionnaire was designed for the purposes of this study, and deviated considerably from the original MEQ (Sutin & Robins, 2007) both in length and content. It has not been validated prior to employment. Further research is needed to confirm its applicability to self-representations, and to improve its psychometric qualities.

Another crucial assumption of CLT is that temporal distance functions as a scale with the present self as a central reference point, meaning that past selves should become more psychologically distant with time. To investigate this, we manipulated temporal distance from the past self to be either close (“yesterday”) or remote (“5 years ago”). We hypothesized that performance would be better for the closer than for the more distant time period, similar to how close others are prioritized over strangers when social distance is manipulated instead. Indeed, we found a modulation of matching performance by remoteness of past time period, such that for the self 5 years ago, there no longer was a significant disadvantage in accuracy compared to a stranger. However, the pattern of this interaction is not consistent with a mediation of perceptual prioritization by temporal distance. First, we observed no effect of temporal distance on performance in the past self condition itself. Descriptively, performance for the temporally close self (“yesterday”) was even poorer than performance for the remote self (“5 years ago”), which runs counter to this hypothesis (see Table 1). Rather, this interaction seems due to opposing effects of the past self’s remoteness on the present self and stranger. No change was predicted for these conditions based on the mediation hypothesis. This interaction needs to be interpreted with caution, however, since Golubickis et al. (2017) did not report effects of temporal distance, and since we did not predict this specific interaction pattern, which was only evident in one of our two dependent variables.

Nevertheless, this preliminary finding suggests an interesting hypothesis concerning the potential impact of both discrimination costs and psychological distance. In our introduction, we discuss the role similarity may have played in prior results regarding past selves in perceptual matching. We suggest that, since Golubickis et al.’s (2017) participants always needed to distinguish between two different types of temporally extended selves, additional discrimination costs for those two conditions may have obscured potential past self-prioritization. Our experiment was designed to address this by presenting participants with a simpler task that did not require this distinction. It remains possible, however, that participants still had to contend with selective discrimination costs which may have differed according to the remoteness of the past self.

Similarity between the distant past self and stranger might account for the lack of difference in error rates between those conditions in the remote group. That is, if the stranger was easier to distinguish from the self yesterday than from the self 5 years ago, one would expect a larger difference between those conditions for the near compared to the remote past self. By the same token, the past self condition also needs to compete with the present self in the matching task, and the self yesterday can be assumed to be more similar to, and less distant from, the present self than the self 5 years ago. In such a case, discrimination costs for the near versus remote past self would differ depending on whether they are relative to a present self or a stranger. In either case, the past self may be considered as more similar to the present self and the stranger conditions than those two referent conditions are to each other, which may explain its relative de-prioritization as the result of additive costs from both competitors.

Note that despite the interaction effect, there was a consistent advantage for the present self over all other referent conditions. This is in line with previous experiments which found self-prioritization to persist even when the label’s distinctiveness was reduced (Schäfer et al., 2017). Regarding the absence of past self-prioritization, it remains possible that there was a residual discrimination cost due to the continued presence of two self conditions. At first glance, this seems implausible, since participants had little difficulty matching the present self-label, and therefore, seemed to have an easy time telling the two conditions apart. However, previous results on the SPE open the possibility that such a discrimination cost could have been asymmetrically distributed, due to a privileged status of the present self condition. In previous experiments on the SPE, the self has been spared from performance costs that affect both close and distant others more strongly (Schäfer et al., 2017; Sui et al., 2012, 2014). If participants were still affected by the additional difficulty of telling apart different selves in our experiment, the cost of this discrimination could have selectively fallen on the past self condition. For instance, competition with the past self may have induced more effective shielding (Fischer et al., 2018) of the present self, which in turn could have caused the past self’s apparent de-prioritization. This robustness of (present) self-prioritization (Schäfer et al., 2017; Sui et al., 2012, 2014) raises the possibility that the relative prioritization of close others is not produced via the same mechanism, as it is comparatively more vulnerable to disruption. This would be consistent with a view of the self as qualitatively “special” (Sui & Humphreys, 2015a; Sui et al., 2012, 2014; Siu & Humphreys, 2013. Such a privileged status of the present self, which is consistently prioritized in cognition, also fits with CLT’s assumption of the present self as the central reference point for mental representations (Trope & Liberman, 2010): The present self is unique in that it is our only source of immediate experience, while neither past selves nor social others can be represented without prior engagement of mental construal processes. Following this line of reasoning, the self’s biasing effect on cognition can be thought of as rather automatic, and as independent of learned contingencies, or available attentional resources (Sui et al., 2012, 2014; Turk et al., 2008).^13^

More generally, similarity-based competition between different conditions might interfere with prioritization effects and vice versa, thereby obscuring either, or both. If perceptual prioritization is based on differences in psychological distance while greater degrees of similarity also lead to larger discrimination costs (Voss et al., 2004), then perceptual prioritization might be masked when sufficiently similar conditions are pitted against each other. Harking back to CLT, similarity is related to psychological distance. For example, it is a crucial determinant of social distance (Liviatan et al., 2008). Equally, two objects could be similar in terms of their distance from the self, occupying the same representational space. In our own experiment, we observed relative prioritization of a stranger over a recent past self, but no difference between a stranger and a more remote past self, the latter of which should be more akin to another person (Pronin & Ross, 2006). If, due to its confounding with similarity, discrimination costs are to some degree coincidental with psychological distance, they could counteract and obscure residual prioritization of past selves. While our design renders such “hidden” prioritization of past selves less plausible than previous experiments, further research could test for this by removing competition between different self conditions within the same task entirely. Furthermore, temporal and social distance should be varied in a more fine-grained manner and independently of each other, and relative similarity and distance between referent conditions could be manipulated systematically. For instance, finer gradation of both psychological distance, and task difficulty could be achieved by including a number of different referents in a between-group or balanced-order design. Psychological distance of a given stimulus can also be controlled for by assessment via self-reports (Broom et al., 2021; Moseley et al., 2021; Sui & Humphreys, 2015b).

One more finding merits attention: since perceptual prioritization effects seem more robust and are more consistently reported for the matching trials (Schäfer et al., 2015; Sui et al., 2012), we did not include a priori hypotheses for the non-matching trials. However, we found a main effect of referent on matching performance in this condition, showing a reversal of the pattern from the matching trials: participants were faster and more accurate when they had to reject the stranger rather than either the present or past self-label. This fits with results reported by Moseley et al. (2021). While they did not observe reduced performance in non-matching trials for the self and a close other (“friend”) in a group of autistic adults, they obtained similar results as ours in a non-autistic control group. Based on the present evidence, the close other condition cannot be considered analogous to a past self in perceptual matching. Interestingly, however, for the non-matching trials, our results for the past self-resemble that for Moseley et al.’s close other. Golubickis et al. (2017), who also investigated effects of temporally extended selves on matching performance, included non-matching trials in their analyses. For error rates, where they pooled matching and non-matching trials together, there was still clear evidence of overall self-prioritization. In reaction times, they observed interactions between referent condition and trial type, which translated into an SPE in matching trials, but not non-matching trials. They did not further break down this interaction between referent and trial type. While descriptively, the advantage for a stranger over the present self in non-matching trials occurs only in two of Golubickis et al.’s (2017) three experiments, their results do not seem inconsistent with ours. Rather, since the effect of referent label in non-matching trials seems smaller than in matching trials, a larger sample might have been required to detect them. Moseley et al. (2021) interpret the reduced performance for the self in non-matching trials in terms of delayed attentional disengagement. Indeed, engagement of attentional resources by self-relevant stimuli is well-documented (e.g. Alexopoulos et al., 2012 Roer & Cowan, 2021). However, it is not clear why facilitation versus disruption should occur at the response (matching versus non-matching), rather than the task level, where facilitatory effects of the self would be predicted for perceptual matching, because the referent label is always task-relevant. Other potential explanations for this finding might include corresponding salience asymmetries between the different referents and response types (Rothermund & Wentura, 2004), or a preference for, or association with affirmative responses regarding the self. However, results from the non-matching trials should be interpreted with caution, as effects appear to be smaller and less consistent, and are not always reported. Theoretical accounts of the SPE often refer to self-prioritization overall or in matching trials only (e.g. Golubickis et al., 2017; Schäfer et al., 2015, 2017). More research is therefore needed to understand response-specific effects in perceptual matching. Due to these considerations, we did not make specific predictions for the non-matching trials, and hypothetical accounts of our results for those trials should be considered exploratory. This is especially true for past self conditions for which little preliminary SPE research exists.

Our findings offer further support for the view that individuals prioritize information related to themselves over information related to others. However, they also appear to imply that this type of prioritization is limited to the present moment, while past selves appear to incur some amount of de-prioritization even relative to a stranger. There remain open questions regarding to which extent task design and competition between experimental conditions may affect matching performance for past selves. Several sound theoretical reasons justify further examination of the extent to which representations of the past self might have unique consequences for information processing. In future studies, the respective effects of social and temporal distance should be varied independently of each other, and carefully controlled.

In light of the conceptual and empirical heterogeneity of research on the self (Klein, 2012; Morin, 2017), it is plausible that psychological distance has differential effects on self-processing in different cognitive domains. For example, the uniqueness of the present self in terms of immediate experience (Trope & Liberman, 2010) may be more relevant to attentional engagement and rapid response selection than for the storage and retrieval of information. It remains unclear if and to what extent retrospective self-reference facilitates explicit memory, a question currently under investigation in our laboratory. To gain a better understanding of the role of psychological distance for cognitive self-biases, self-prioritization should be compared to other paradigms and outcome measures using analogous manipulations.

## Data Availability

Experimental data (stripped of identifying information) are available in the supplementary materials, as well as directly from the corresponding author. Please understand that for privacy reasons, we are not sharing the data from the demographic questionnaire in the supplement. The experiment file is available via the Labvanced platform (link: https://www.labvanced.com/page/library/9822; requires account) or upon request. A print version of the demographic questionnaire (German language) is available upon request.

## Appendix 1: Self-report questionnaire

See Table 3.

**Table 3:** Questionnaire items used in our study with English translations, their corresponding subscale (including number of items and internal consistencies as indicated by Cronbach’s α), and, where applicable, the corresponding MEQ item (see Sutin & Robbins, 2007, p. 410f) used as a reference point

Subscale	No	Item	Translation	MEQItem
Vividness *n* = 4 *α* = .82	1	Die Vorstellung von mir selbst zu dieser Zeit war lebendig	My image of myself during this time was vivid	1.2
	3	Meine Erinnerung an diese Zeit war klar	My memory for this time was clear	1.1
	4	Meine Erinnerung an diese Zeit war lebhaft	My memory for this time was vivid	1.2
	5	Meine Erinnerung an diese Zeit war vage (R)	My memory for this time was vague	1.5
Accessibility *n* = 2 *α* = .74	15	Ich musste mein Gedächtnis wirklich durchsuchen, um mich in diese Zeit zurück zu versetzen (R)	I really had to search my memory to think back on this time	3.5
	20	Es war einfach, mich an diese Zeit zurück zu erinnern (R)	This time was easy for me to recall	3.2
Valence *n* = 4 *α* = .73	6	Meine Erinnerung an diese Zeit war im Großen und Ganzen positiv	My memory for this time is mostly positive	10.1/10.2
	12	Als ich mich an diese Zeit zurückerinnerte, mochte ich die Person, die ich zu dieser Zeit war	When thinking back on this time, I liked the person I was then	n/a
	17	Die Person, die ich zu dieser Zeit war, war mir unsympathisch (R)	I do not like the person I was during this time	n/a
	19	Meine Gefühle zu dieser Zeit waren vorwiegend negativ (R)	My feelings during this time were mostly negative	10.6
Distancing *n* = 2 *α* = .66	10	Als ich mich an diese Zeit zurückerinnerte, hatte ich das Gefühl, die Person aus dieser Zeit war dieselbe Person wie ich heute (R)	When thinking back on this time, I felt like I was the same person then that I am today	9.5
	14	Als ich mich an diese Zeit zurückerinnerte, hatte ich das Gefühl, die Person aus dieser Zeit sei eine andere Person als ich heute	When thinking back on this time, I felt like the person from this time is a different person than who I am today	9.2
Emotional Intensity *n* = 2 *α* = .60	2	Die Vorstellung an diese Zeit berührte mich emotional	Imagining this time touched me emotionally	n/a
	7	Als ich mich an diese Zeit zurückerinnerte, hatte ich keine starken Emotionen in Bezug auf diese Zeit (R)	When thinking back on this time, I did not have strong emotions about this time	5.5
Visual Perspective *n* = 3 *α* = .42	13	Als ich mich an diese Zeit zurückerinnerte, kam esmir so vor, als würde ich mich selbst von außen beobachten (R)	When thinking back on this time, I felt like I was an observer watching myself	6.6
	16	Ich erlebte die Erinnerung an diese Zeit durch meine eigenen Augen	I saw the memory of this time through my own eyes	6.1
	18	Als ich mir diese Zeit vorstellte, sah ich die Welt eindeutig aus der Ich-Perspektive	When visualizing this time, I clearly saw the world from my own perspective	6.3
Sensory Detail *n* = 3 *α* = .27	8	Als ich mich an diese Zeit zurückerinnerte, konnte ich mich selbst körperlich in dieser Zeit spüren	When thinking back on this time, I could bodily feel myself in this time	4.3
	9	Als ich mich an diese Zeit zurückerinnerte, fühlte essich nicht wirklich so an, als würde ich sie wieder erleben (R)	When thinking back on this time, it did not really feel like I was reliving it	4.6
	11	Als ich mich an diese Zeit zurückerinnerte, erlebte ich dieselben Gefühle, die ich zu dieser Zeit hatte	When thinking back on this time, I could feel the same emotions that I felt then	4.2

## Appendix 2: Matching performance means for all conditions

See Table 4.

**Table 4:** Mean reaction times (in ms) and accuracies (in %) as a function of remoteness group (near past/ “yesterday” and remote past/“5 years ago”, reminiscence group (instructions and no instructions), referent label (current self, past self and stranger) and trial type (matching and non-matching)

Group: reminiscence	Group: remoteness	Person label	*N*	Reaction time (ms)		Accuracy (%)	
				Matching	Non-matching	Matching	Non-matching
Instructions	Near past	Self now	29	532 (90)	652 (101)	.87 (.16)	.76 (.14)
		Self yesterday		631 (116)	650 (110)	.72 (.15)	.76 (.15)
		A stranger		600 (113)	630 (105)	.80 (.13)	.85 (.11)
	Remote past	Self now	31	544 (97)	662 (125)	.90 (.09)	.74 (.16)
		Self 5 years ago		624 (122)	666 (115)	.75 (.15)	.73 (.14)
		A stranger		612 (115)	640 (114)	.76 (.14)	.82 (.17)
No Instructions	Near past	Self now	29	545 (112)	659 (121)	.84 (.19)	.73 (.19)
		Self yesterday		607 (146)	649 (123)	.73 (.17)	.75 (.16)
		A stranger		576 (117)	636 (114)	.80 (.11)	.85 (.12)
	Remote past	Self now	29	561 (118)	675 (127)	.88 (.11)	.72 (.18)
		Self 5 years ago		639 (115)	677 (123)	.75 (.13)	.74 (.15)
		A stranger		615 (128)	658 (111)	.77 (11)	.85 (.11)

SD in parentheses

## Footnotes

1 This is assuming that the experiments were adequately powered to detect past self-prioritization. While they would likely have obtained reliable condition means from each participant due to the large number of trials, their sample size of *N* = 16 participants for each experiment can be considered rather small.

2 To our knowledge, the only published study involving temporally distant selves is the one by Golubickis et al. (2017). However, we think that using their results as input for our power analysis would have been problematic, since their design and hypotheses differ from ours in important ways. For example, while they report no prioritization of past selves regardless of remoteness, we predict that some prioritization might occur in a simplified design.

3 To detect a 2 × 2 interaction (e.g. a modulation of the difference between two referent conditions by temporal distance) with the same power as a corresponding main effect, at least twice the number of observations are required (Simonsohn, 2014) resulting in a minimum desired sample size of *N* = 118.

4 Since conventionally, intercorrelations are set at .5, this serves to make our estimate more conservative.

5 Given the typically robust effects found in the paradigm together with findings showing the general validity of online experiments (Semmelmann & Weigelt, 2017) as well as our own experience with the successful implementation of online studies (e.g. Beckman et al., 2019), we consider this setting suitable for an SPE experiment.

6 During the experiment, pictures were resized to correspond to a frame of 100 × 100 internal Labvanced design units, which were then rescaled according to participants’ monitor size. Given the expected variability of hardware parameters and informal testing situation, neither visual angle nor absolute stimulus size were kept constant for. For example, on a 13′3-inch screen, the square shape would be 4cmx4cm in height and width. While this is a potential source of noise, there is no substantive reason to assume the SPE would depend on such presentation parameters. In fact, such a susceptibility would present a serious problem for its interpretation.

7 This comparatively long fixation period was chosen to avoid responses to the previous stimulus pair being recorded as a response for the current trial. This precaution was taken after piloting revealed that some volunteers had difficulty observing the deadline.

8 These rather stringent exclusion criteria were chosen a priori, with the goal of ensuring adherence to task instructions in our sample despite the lack of laboratory conditions — performing ancillary analyses including the other 14 participants who completed the experiment did not alter the pattern of results.

9 This means that some responses were included despite being made after the response deadline set by the experimenter. Since both conventionally and theoretically, we saw no reason to consider these responses as originating from a distinct process or distribution than those of interest to us, and since those late responses do not overlap with those for the following trial, we opted not to remove them.

10 But see also Moseley et al. (2021), and Golubickis et al. (2017) who included trial type (matching vs. non-matching) as a within-subjects factor. We consider potential interpretation of non-matching trials in the discussion.

11 As described in the method section, our questionnaire differed substantially from the original version of the MEQ and only contained 20 items. This means that for each subscale, there was a lower number of items in our analogue than there are in the MEQ, and that the number of items varied between subscales.

12 We discuss the single exception to this pattern further below.

13 Even so, upon closer inspection, we found an interaction that hinted at a slight impairment in accuracies for the present self, when it had to be distinguished from a temporally close, rather than a temporally distant self. While this specific finding was unexpected and requires additional empirical confirmation, it leaves open the possibility that the present self is somewhat more vulnerable to interference than initially assumed, and that a sufficiently “close” competitor (e.g. self yesterday) can interfere with present self-matching.
